# Crystallographic control of the fabrication of an extremely sophisticated shell surface microornament in the glass scallop *Catillopecten*

**DOI:** 10.1038/s41598-022-15796-1

**Published:** 2022-07-07

**Authors:** Antonio G. Checa, Carmen Salas, Francisco M. Varela-Feria, Alejandro B. Rodríguez-Navarro, Christian Grenier, Gennady M. Kamenev, Elizabeth M. Harper

**Affiliations:** 1 Department of Stratigraphy and Paleontology, University of Granada, 18071 Granada, Spain; 2 Instituto Andaluz de Ciencias de La Tierra, CSIC-University of Granada, 18100 Armilla, Spain; 3 Department of Animal Biology, University of Málaga, 29071 Málaga, Spain; 4 Centro de Investigación, Tecnología e Innovación, University of Sevilla, 41012 Sevilla, Spain; 5 Department of Mineralogy and Petrology, University of Granada, 18071 Granada, Spain; 6 A.V. Zhirmunsky National Scientific Center of Marine Biology, Far Eastern Branch, Russian Academy of Sciences, Vladivostok, 690041 Russia; 7 Department of Earth Sciences, University of Cambridge, Cambridge, CB2 3EQ UK

**Keywords:** Biophysics, Structural biology, Solid Earth sciences, Materials science

## Abstract

The external surface microornament of the glass scallops *Catillopecten natalyae* and *malyutinae* is made by calcitic spiny projections consisting of a stem that later divides into three equally spaced and inclined branches (here called aerials). *C. natalyae* contains larger and smaller aerials, whereas *C. malyutinae* only secreted aerials of the second type. A remarkable feature is that aerials within each type are fairly similar in size and shape and highly co-oriented, thus constituting a most sophisticated microornament. We demonstrate that aerials are single crystals whose morphology is strongly controlled by the crystallography, with the stem being parallel to the *c*-axis of calcite, and the branches extending along the edges of the {104} calcite rhombohedron. They grow epitaxially onto the foliated prisms of the outer shell layer. The co-orientation of the prisms explains that of the aerials. We have developed a model in which every aerial grows within a periostracal pouch. When this pouch reaches the growth margin, the mantle initiates the production of the aerial. Nevertheless, later growth of the aerial is remote, i.e. far from the contact with the mantle. We show how such an extremely sophisticated microornament has a morphology and co-orientation which are determined by crystal growth.

## Introduction

The way in which bivalved molluscs secrete their shells has been a topic of intense interest^1–4^. This research has revealed a universal process whereby shell construction follows a series of consecutive stages: (1) secretion of a sheet of largely organic periostracum within the periostracal groove^2–7^, which (2) is extruded, sliding along the internal face of the most external fold of the mantle, the outer mantle fold (OMF); (3) the periostracum curves back at the distal end of the OMF and (4) secretion of the mineral shell layers begins underneath^3^. Although data are limited, the shell growth surface appears to be in close contact with the external surface of the OMF, leaving a negligibly thin extrapallial space in between in the taxa investigated^8–10^. Some bivalves, however, modify this basic model so that mineralization initiates within the periostracum before a continuous shell begins to forms. This intraperiostracal mineralization has so far been described in a range of taxa and showing a variety of forms, for example, spikes or plaques in anomalodesmatans, gastrochaenids and trigoniids^10–12^, needles in venerids^13^ or granules in certain mytilids^14^ which project above the valve surface as part of a shell ornament. Despite this diversity, these intraperiostracal growths have relatively simple morphologies, all appear to be composed of aragonite and are initiated in the innermost translucent layer of the periostracum^10–12^. Whereas in some, for example, the granules in mytilids^14^ and needles in venerids^13^, the intraperiostracal mineralizations are entirely separate from the main body of the calcified shell, in most instances studied they are fully continuous with it^10–12^.

**Figure 1:**
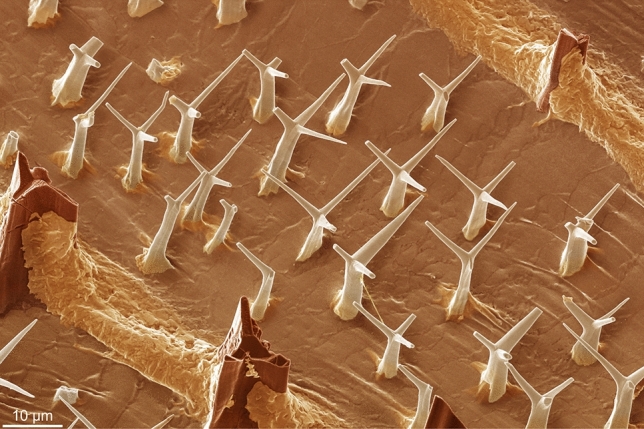
View of the shell surface of *Catillopecten natalyae*, decorated with low order aerials with their characteristic three branched morphology occupying the area between two ribs. The aerials are all cooriented and nearly evenly spaced. SEM coloured image, where the different structures (high-, low-order aerials and secondary deposits) have been applied different colors. See also Supplementary Fig. S2.

This paper investigates the intriguing case of surface microornament of two species of the propeamussiid *Catillopecten*, *C. malyutinae* Kamenev, 2017, and, especially, *C. natalyae* Kamenev, 2017, recently described by Kamenev^15^ from the abyssal plain of the NW Pacific. The Propeamussidae, colloquially known as glass scallops, often live at bathyal and abyssal depths and, consequently, despite the number of taxa, aspects of their biology are not well known. All have the ability to swim^16^ and a number of species are known to be carnivorous^17–19^. Unlike most members of the Propeamussiidae which generally have smooth shells, the surfaces of both right and left valves of *C. natalyae* and *C. malyutinae* are studded with regularly-shaped minute intricately shaped elements consisting of a vertical stem that trifurcates into three equally-angled branches (Fig. 1). Henceforth we will call them aerials on account of their superficial similarity to TV or radio aerials. They appear to represent the height of morphological sophistication in terms of shell ornament. In the original description^15^, these structures were referred to as periostracal setae. However, these aerials with their marked geometric morphology which recall crystallographic ‘design’ and their breakage pattern appear to be mineralized. They are much more sophisticatedly shaped than any other intraperiostracal structure described to date, or indeed any external microornament in general and it is intriguing how they can attain such intricate geometric shapes seemingly far from the contact with the mantle.

Our purpose here is to (1) fully characterize the morphology and crystallography of the aerials, (2) elucidate the relationship to the rest of the shell, (3) propose a mechanism for their formation.

## Results

### General morphology

Detailed descriptions of the overall morphology of both species are given in ^15^. Both are small-sized, with the specimens analysed here all being less than 8 mm in dorsoventral diameter. The valves themselves are very thin (around 10–20 μm) and translucent, and have the typical D-shape of scallops with relatively large auricles (Supplementary Fig. S1). Both species have a prominent byssal notch on the right valve.

The aerials are present on both valves though more developed on the left valve that is uppermost in life. In *C. natalyae* there are two different types of aerials, and only one in *C. malyutinae.* The details are given below.

**Figure 2:**
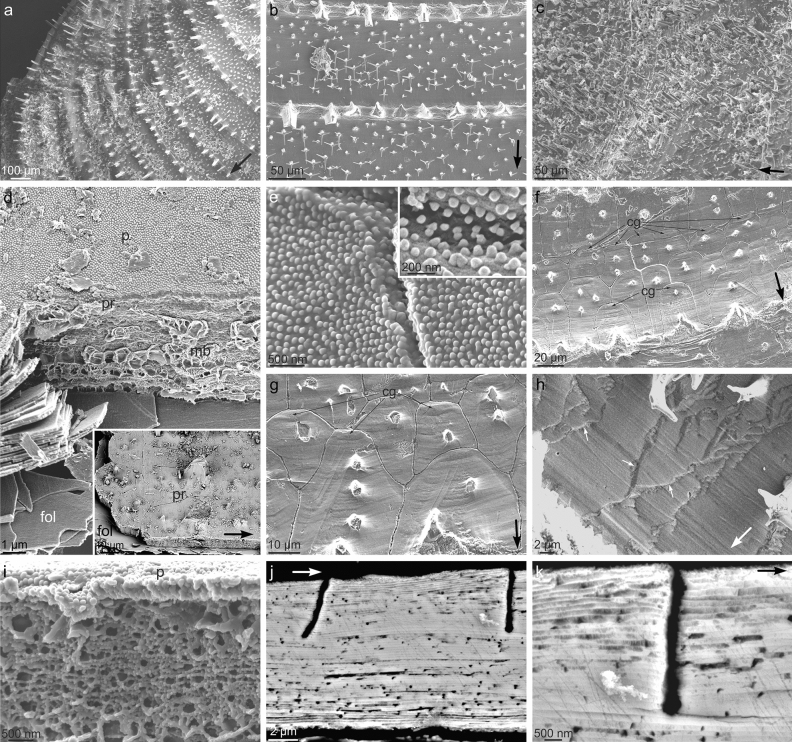
Shell structure of *Catillopecten*. (**a**) General view of the shell outer surface of *Catillopecten natalyae* showing the ornament of commarginal ribs and first- and second-order aerials. (**b**) Detail of the first-order aerials of *Catillopecten natalyae* (broken) protruding from the ribs, and the second-order aerials populating the inter-rib space with a quasiperiodic distribution. (**c**) Aspect of the evenly sized aerials of *Catillopecten malyutinae*. (**d**) Close up and general view (inset) of the prismatic (pr) and foliated (fol) layers of *Catillopecten natalyae*. The periostracum (p) covering the prisms and the interprismatic membrane (mb) are also discernible. The set of laths on the left side results from the dismantling of one of the prisms. (**e**) Detail of the periostracum of *Catillopecten natalyae*. It is characterized by a surface ornament of tiny pimples with slightly conical outlines (inset). (**f**), (**g**) Surface view of the prismatic layer of *Catillopecten natalyae*. Note the convex adoral outlines of the prisms. The centres of growth (cg) of some of them are indicated. (**h**) Surface view of an area of *Catillopecten natalyae* where prisms have been replaced by irregular granules elongated in the growth direction (some outlines are indicated with small arrows). (**i**) Detail of the vacuolated membranes of *Catillopecten natalyae* delineating the individual prisms. The periostracum (p) is to the top. (**j**), (**k**) Two longitudinal sections of *Catillopecten malyutinae* at different magnifications across the shell. The prisms are made by exactly the same foliated material as the underlying foliated layer. The black slits correspond to the positions of the membranes. The big arrows in (**a**) to (**c**), (**d**) inset, (**f**) to (**h**), (**j**) and (**k**) indicate the growth direction of the shell.

The external ornamentation of *C. natalyae* consists mainly of commarginal ribs, which are continuous all along the margin with spacing of 40–150 μm, being more widely spaced in the maximum growth direction (Fig. 2a, b, and Supplementary Fig. S1a). Wide, low relief commarginal ribs are also present in *C. malyutinae* (Supplementary Fig. S1b). Besides the ribs, the surface is studded with projections in the form of the roughly perpendicular aerials (Figs. 1, and 2b, c) that constitute the main subject of this study. In *C. natalyae t*here are two orders of aerials: (1) Large aerials, protruding from commarginal ribs and (2) smaller aerials, in the inter-rib areas that form dense aggregations of more or less co-oriented elements (Figs. 1 and 2b). The larger aerials are not present in *C. malyutinae* (Fig. 2c)*.* They are described in detail below.

### General valve microstructure

The shell of both species is made of an outer periostracum, underlain by an outer calcitic prismatic layer and an inner foliated layer (Fig. 2d). The latter layer extends to the position of the myostracum. We have no data on the distribution of more internal layers but details are given for the family in general by Waller^16^.

The whole shell surface, including the ribs and aerials, is covered by the periostracum. It is typically studded with circular pimples with radii of 50–60 nm and spaced at ca. 150 nm. They are slightly higher than wider and seem to have slightly conical shapes (Fig. 2d, e). The aspect of the periostracum of *C. malyutinae* under the TEM is described below.

**Figure 3:**
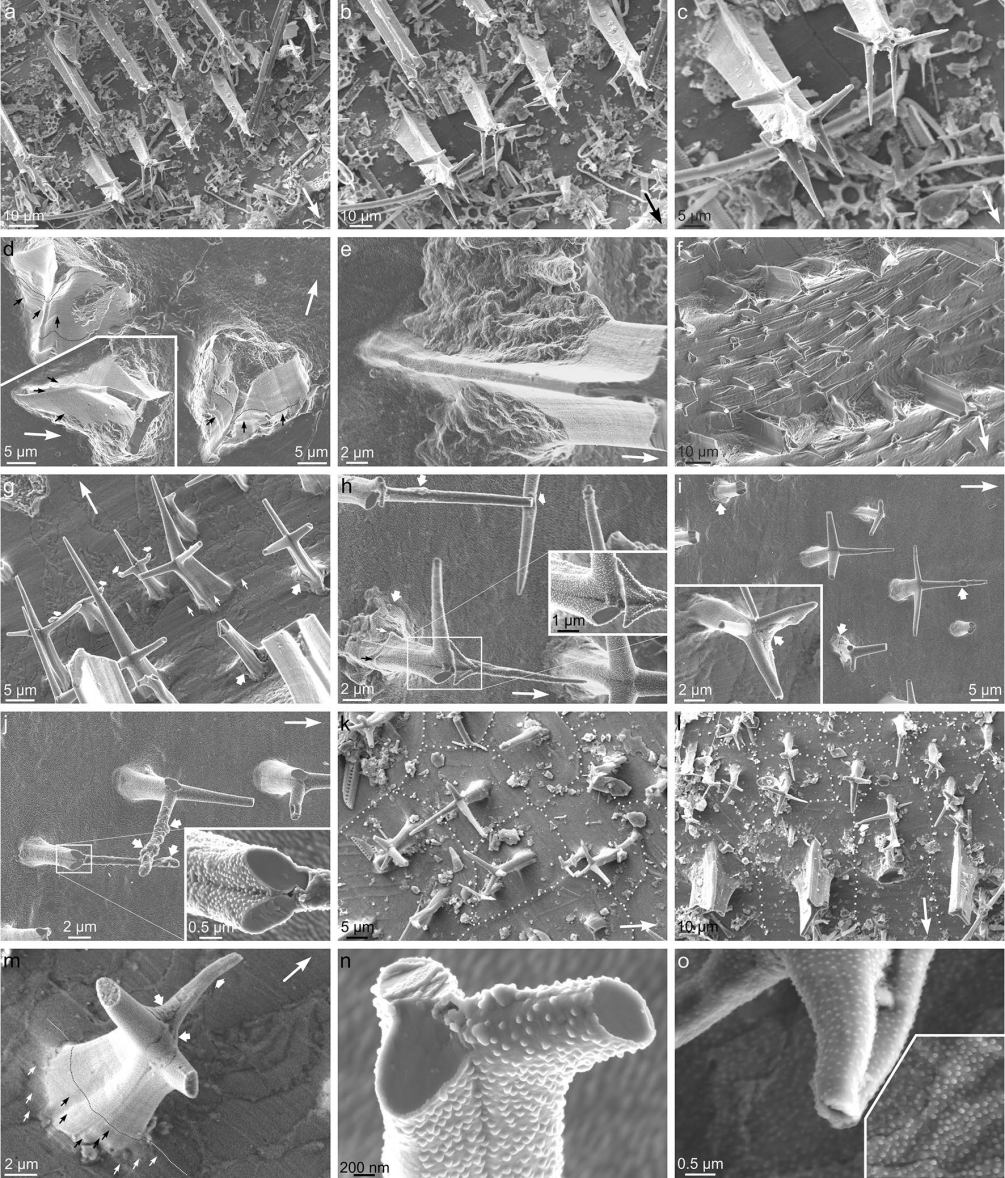
External morphology and distribution of first- and second-order aerials of *Catillopecten natalyae.* (**a**)–(**c**) Extremely well preserved first-order aerials showing the arrangement of evenly distributed spikes at 120° (in top view), following three-fold symmetry. (**d**) Distribution of growth lines (small arrows) close to the base of broken first-order aerials. Some growth lines have been outlined with broken lines. Note irregular additional deposits at the sides of the aerials. (**e**) Detail of the base of a first-order aerial surrounded by secondary deposits. Growth lines are also visible on the aerial. (**f**) General view of two ribs and the inter-rib space, together with the accompanying first- and second-order aerials, respectively. There are abundant periostracal wrinkles. (**g**) Oblique view of second-order aerials. Some of them show secondary deposits at their bases (intermediate arrows). The short wide arrows indicate bent branches, and the thin arrows point to growth lines. (**h**) Details of second-order aerials showing consistent smooth fractures at high angles to the branch axes (inset). Note duplicate branches in the framed aerial. Arrows indicate as in **g**. (**i**), (**j**) Set of second-order aerials showing secondary deposits both at their bases and branches (intermediate arrows). Note smooth fractures at consistent angles to the branches in **j** (inset). (**k**), (**l**) Top views of groups of aerials. Note strict co-orientation of branches when aerials settle on the same prism (prism outlines indicated with loosely dotted lines). The short wide arrow in **l** indicates a bent branch. (**m**) Aerial with well-defined secondary deposits (thick arrows) and growth lines (thin arrows, and broken line). The short wide arrow points to a bent branch. The shell surface displays outlines of irregular elongated grains. (**n**) Detail of an aerial showing smooth fractures at consistent inclinations and orientations with respect to the branch axes. (**o**) Details of the tip of a branch and the underlying shell surface (inset). Note marked difference in the density of periostracal pimples. The long arrows in (**a**) to (**m**) indicate the growth direction of the shell.

The prismatic layer is made of flat prisms (4–5 µm thick) with variable widths (15–30 µm) (Fig. 2d, and inset, f, g, j). At the positions of the ribs, they are particularly wide (up to 60 µm) and long (up to 100 µm). They invariably have convex dorsal boundaries and their relationship with the adoral and adapical neighbours is random, i.e., they can be aligned or offset to any degree (Fig. 2f, g). The growth lines, when observable, are parallel to the shell margin and run uninterruptedly across the different prisms (Fig. 2f, g). When visible, the centre of growth of prisms is marked by concentric oval (commarginally elongated) growth lines, which initiate close but separate from the apical end (Fig. 2f, g). In some areas of the shell surface, the prismatic outlines are not visible and are replaced by structures with very dissimilar shapes and sizes (1–10 µm in commarginal dimension) usually elongated radially (up to several tens of µm) (Figs. 2h, and 3g, m). The boundaries between prisms are marked by thin organic membranes which have vacuolar nature (Fig. 2d, i) and are only a few hundred nanometers wide (Fig. 2j, k). Both in fracture and sections, it is observed that the prismatic and the underlying foliated layers are made by exactly the same material: very thin calcite laths (~ 150 nm), subhorizontal or at a low angle to the surface (Fig. 2j, k). The only difference between the outer prismatic and the inner foliated layers is in the organic membranes delineating the prisms that penetrate some 5–6 µm (Fig. 2d, i–k).

### Details of aerials

The larger first-order aerials of *C. natalyae* are fairly evenly distributed along the commarginal ribs (every 40–60 µm) (Figs. 2a, b, and 3a–c). They are spiny projections and have tri-radiate sections with the lateral flanges at 120° to one another, (Figs. 1, 2b, and 3a–g, and Supplementary Fig. S2). There is a high, though not strict, degree of co-orientation of the first-order aerials, since there is always one of the flanges pointing towards the apex of the shell (Figs. 1, 2b, and 3a–f, and Supplementary Fig. S2). In some cases of exceptional preservation there is a pair of spikes diverging from every flange, at a high angle from the elevation and forming 120° (in top view) with the flange (Fig. 3a–c). In this way, every aerial contains ideally three pairs of spikes parallel in pairs. This branching process may happen more than once along length of the flange (Fig. 3a–c). Although it is difficult to estimate precisely, some complete first-order aerials may reach around 100 μm in height (Figs. 2a, and 3a–c). The stems of these aerials display closely spaced growth lines seemingly parallel to the shell surface (Fig. 3c–e). Close to the base, in the ventral direction, they dip toward the shell surface and continue into the growth lines of the shell surface (Fig. 3d). The concentric ribs consist mainly of commarginal elevations draped between the bases of neighbor first-order aerials (Figs. 1, 2b, and 3e, f, and Supplementary Fig. S2). Their boundaries are easy to trace since their surfaces are highly irregular and lack growth lines, in contrast to the surfaces of the aerials and of the contiguous prisms (Figs. 1, and 3d–f, and Supplementary Fig. S2). They are clearly secondary deposits formed below the periostracum. These drapes constitute most of the concentric rib elevations. Otherwise, the concentric ribs consist only of relatively shallow undulations (Fig. 2f). In these instances, conspicuous wrinkles on the shell surface, clearly formed in the periostracum, may be observed (Fig. 2f, g).

The inter-rib spaces are studded with much smaller (15–20 μm high), second-order aerials (Figs. 1, 2a, b, and 3g–m, and Supplementary Figs. S2, and S3a). Multiple aerials may be associated with a single prism (Fig. 2g). Their stems are slightly conical, reducing in width with height, and split into three highly-inclined spike-like branches spaced at equal angles, similar to those of the first-order aerials, but relatively longer compared to the reduced height of the second-order aerials (Figs. 1, 2b, and 3g–m, and Supplementary Figs. S2, and S3a). The branches are straight, although, they may curve (Figs. 1, and 3g, h, l, m, and Supplementary Figs. S2, and S3a), sometimes by interaction with those of neighbouring aerials (Figs. 3g, h, and Supplementary Fig. S3a). Duplicate or accessory branches may occur (Figs. 3h, and Supplementary Fig. S3a). Occasionally, the bases of aerials (Fig. 3g–i), and, more rarely, the branches (Fig. 3h, i, and inset, j, m), become buttressed by sub-periostracal deposits with irregular surfaces, similar to those of the drapes found at the base of the first-order aerials. Second-order aerials also display a high co-orientation. There is always a branch pointing in the growth direction, and the range of dispersion within groups is usually less than 20°, exceptionally up to 40° (Figs. 1, 2b, and 3g-l, and Supplementary Figs. S2, and S3a). When two or more aerials grow from a single prism their orientation is identical (Fig. 3k, l). Their delicate nature means that the aerials are frequently broken to different extents (e.g. Figures 1, 2f, g, and 3g, h, j, and Supplementary Fig. S2). The branches are sometimes broken along smooth planes with similar inclinations and orientations with respect to their axes, in a ternary symmetry (Fig. 3h, j, n). The periostracum covers the whole of the aerials, including the most distal tips (Fig. 3g–j, m–o, and Supplementary Fig. S3). Very faint growth lines, more or less parallel to the outer shell surface, are imprinted on the stems, particularly close to their bases (Figs. 3g, h, m, and Supplementary Fig. S3a). As with first-order aerials, the growth lines dip in the growth direction to continue into the growth lines of the shell surface. As a rule, the density of periostracal pimples decreases from the shell surface toward the tips of the branches (Figs. 3o, and Supplementary Fig. S3). Periostracal wrinkles may also develop associated with their bases (Figs. 1, and 3g, and Supplementary Fig. S2).

**Figure 4:**
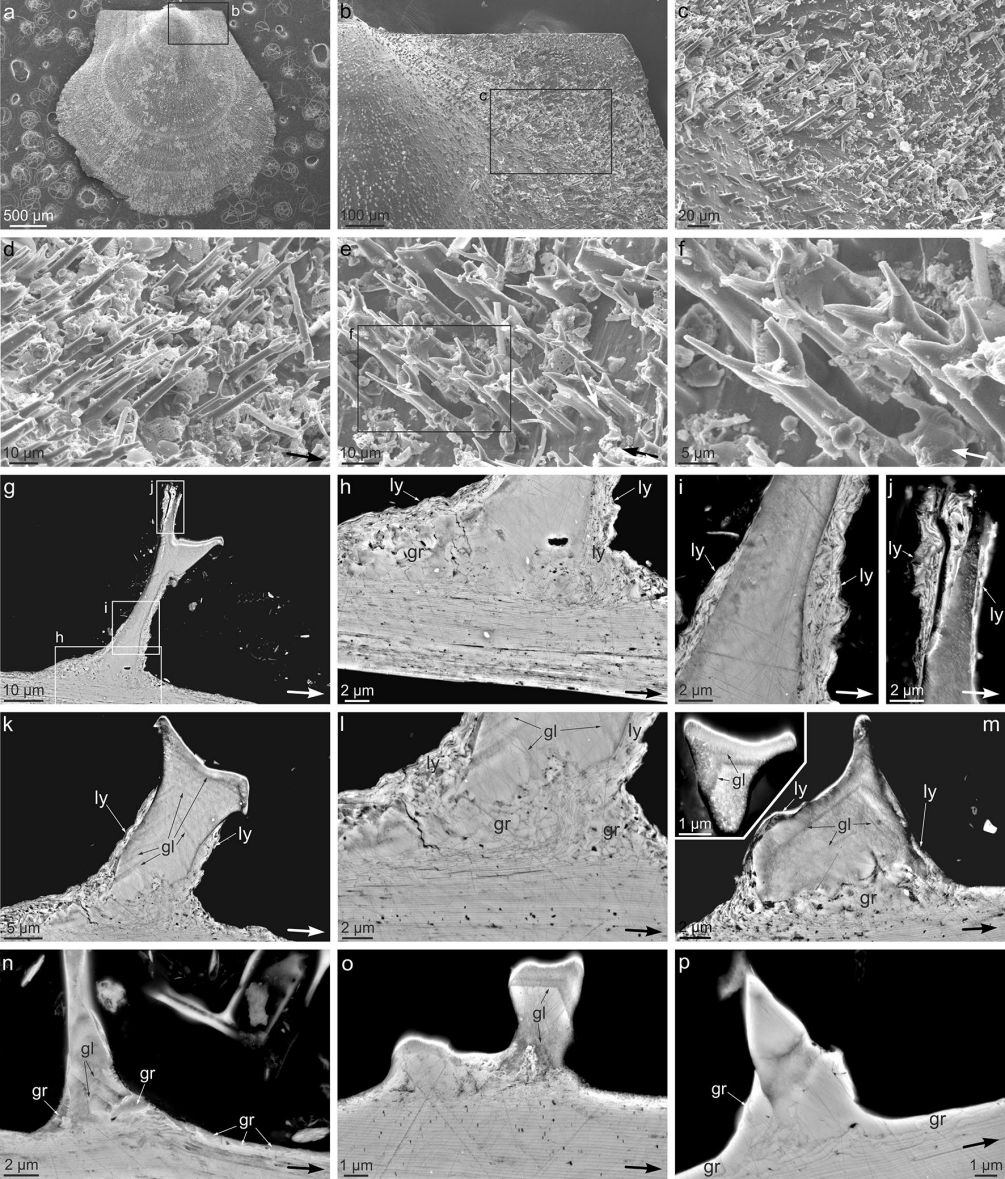
Surface and cross sectional views of the shell and aerials of *Catillopecten malyutinae*. (**a**) General view of a left valve. (**b**), (**c**) Progressive close ups of the posterior area of **a**, showing the distribution of aerials. (**d**)–(**f**) Details of the aerials of the anterior (**d**) and posterior shell areas (**e**), (**f**). Note that there is always a branch pointing in the local growth direction. **b**, **c**, and **f** are close ups of the areas framed in (**a**), (**b**) and (**e**), respectively. The big arrows in **c** to **f** indicate the local growth direction of the shell. (**g**)–(**j**) (**g**) is a general view of an aerial and its insertion within the shell, with indication of the details shown in (**h**) to (**j**) (frames). (**h**) Detail of the contact between the foliated layer and the massive monocrystalline core of the aerial. There are granular (gr) and layered (ly) intervening deposits. (**i**), (**j**) Middle and top parts of the aerial, respectively. Note layered deposits (ly) surrounding the massive monocrystalline core. (**k**)**–**(**p**) Details of other aerials and their contacts with the foliated shell. There are visible growth lines (gl) in (**k**) to (**o**), lateral layered deposits (ly) in (**k**) to (**m**), and granular deposits (gr) in (**l**) to (**n**) and (**p**). These extend onto the shell surface in (**n**) and (**p**). Arrows in (**g**) to (**p**) indicate the shell growth direction in all cases. Images in (**g**) to (**p**) have been made using the SEM backscatter (CBS) detector.

The aerials of *C. malyutinae* are similar in size to those of the second-order type of *C. natalyae*, occurring on both valves and arranged in anti-marginal rows (Fig. 4). They are more strongly developed on the auricles^15^ (Fig. 4a–c). They differ from those of *C. natalyae* by having shorter, less well developed branches (Fig. 4d–f). These tend to curve upward to become increasingly (sometimes strictly; Fig. 4d) parallel to the stem. As in *C. natalyae*, they are co-oriented, with one of the branches pointing in the local growth direction (Fig. 4d–f). The shortage of material has prevented us from doing more detailed surface observations.

In section, aerials of *C. malyutinae* display a massive interior that connects basally with the laminae of the outer shell layer (Fig. 4g, h, k–p). At the contact, the laminae appear highly disturbed and tend to surround the base of the aerials (Fig. 4h, k–m). In some instances, they extend along the sides of the aerial and form a kind of crust (Fig. 4g, h, k–m), which, eventually, reaches the top of the aerial (Fig. 4g, i, j). Occasionally, granular features are also found between the basis of the massive interior of the aerial and the laminated crust (Fig. 4g, h, l–n, p), and may extend onto the shell surface (Fig. 4n, p). The aerial interior frequently contains series of parallel growth lines that appear in different contrast. They tend to form angular outlines with the apex to the top (Fig. 4k–o) though with growth they sometimes flatten approximately perpendicular to the aerial axis (Fig. 4m inset, o).

Under the atomic force microscope (AFM), the same polished sections reveal that both the foliated layer (Supplementary Fig. S4a) and the aerials (Supplementary Fig. S4b) display the nanoroughness typical of biominerals, with similar nanounit size.

### Optical and transmission electron microscopy of the incipient periostracum and periostracal groove of *C. maluytinae*

**Figure 5:**
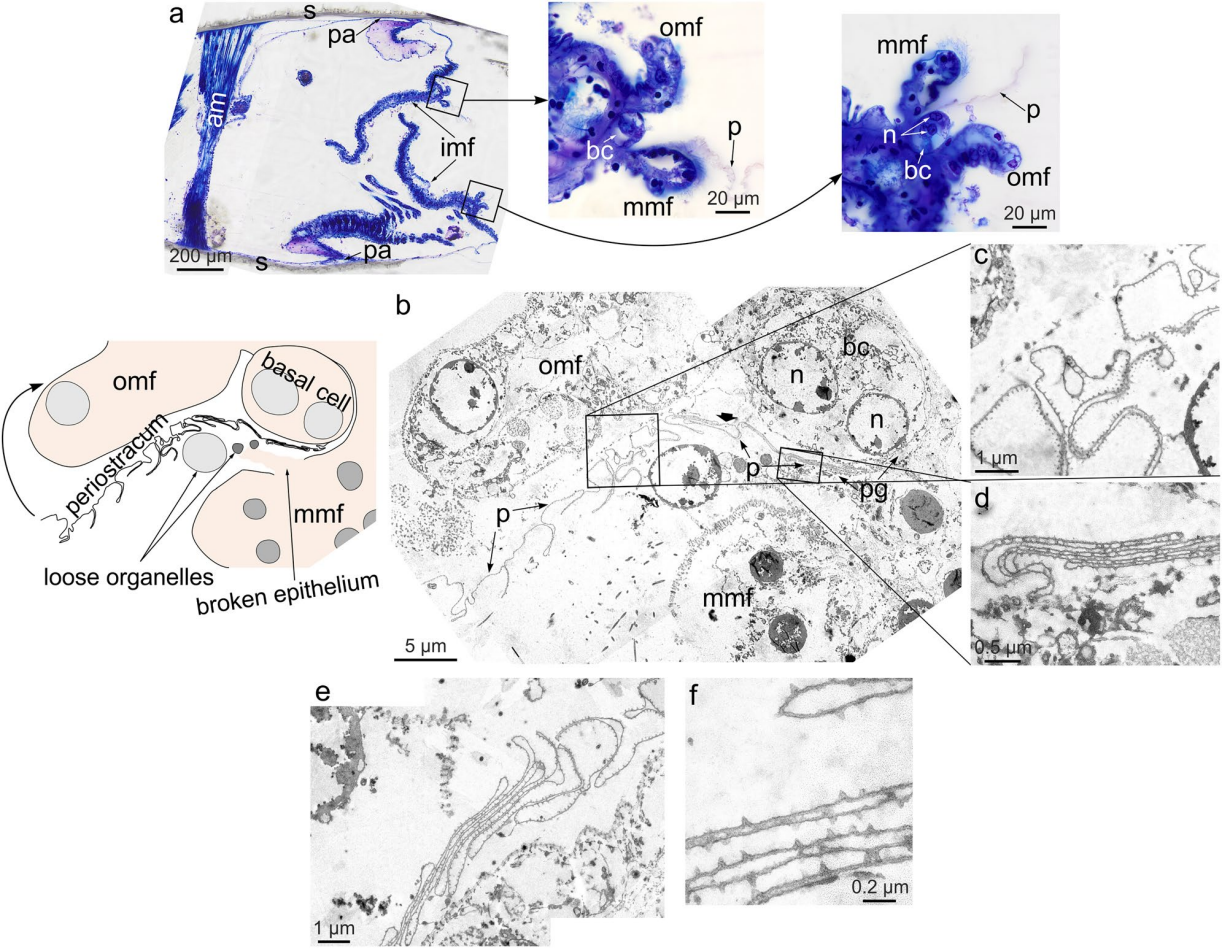
Formation of the periostracum in *Catillopecten malyutinae* studied by optical and electron (TEM) microscopy. (**a**) Thin section of a methacrylate embedded specimen. The close ups correspond to both mantle margins, with indication of the mantle folds, periostracum and basal cell. (**b**) General view (composite image) of the periostracal secretion system. The periostracum is extruded from a binucleate basal cell located at the bottom of the periostracal groove. This is formed between the middle and outer mantle folds. The TEM image is explained in the left sketch (the curved arrow indicates the progression of the periostracum, toward the outer surface of the outer mantle fold). (**c**)**–**(**e**) Details of the heavily folded periostracum within the periostracal groove. (**f**) Close up view of the periostracum with pimples pointing toward the exterior with pointed outlines. *am* adductor muscle, *bc* basal cell, *imf*, *mmf*, *omf* inner, middle, outer mantle fold, *n* nucleus, *p* periostracum, *pa* pallial attachment, *pg* periostracal groove, *s* shell.

The formation of the periostracum has been studied in only a single specimen of *C. malyutinae*, due to shortage of material, and the non-ideal preservation has limited the precision available. We have recognized the three lobes, outer, middle and inner, typical of the mantle margin of most bivalves (Fig. 5a). The periostracum is extruded from a seemingly binucleate basal cell located at the base of the periostracal groove, between the outer and middle mantle folds (Fig. 5a, b). The periostracum appears very heavily folded along the length of the periostracal groove (Fig. 5c–e). The folds are irregular in morphology, but the amplitude remains similar. In detail, the periostracum is very thin (~ 30 nm in TEM sections) and does not change along the length of the periostracal groove. Its outer face is adorned by tiny pimple-like protrusions, which, in TEM section, are taller (70–80 nm) than wider (30–40 nm) and have angular outlines (Fig. 5f). We have not been able to discern any internal structure within the periostracum.

### Crystallography of the shell and second-order aerials by EBSD and TEM of *C. natalyae*

**Figure 6:**
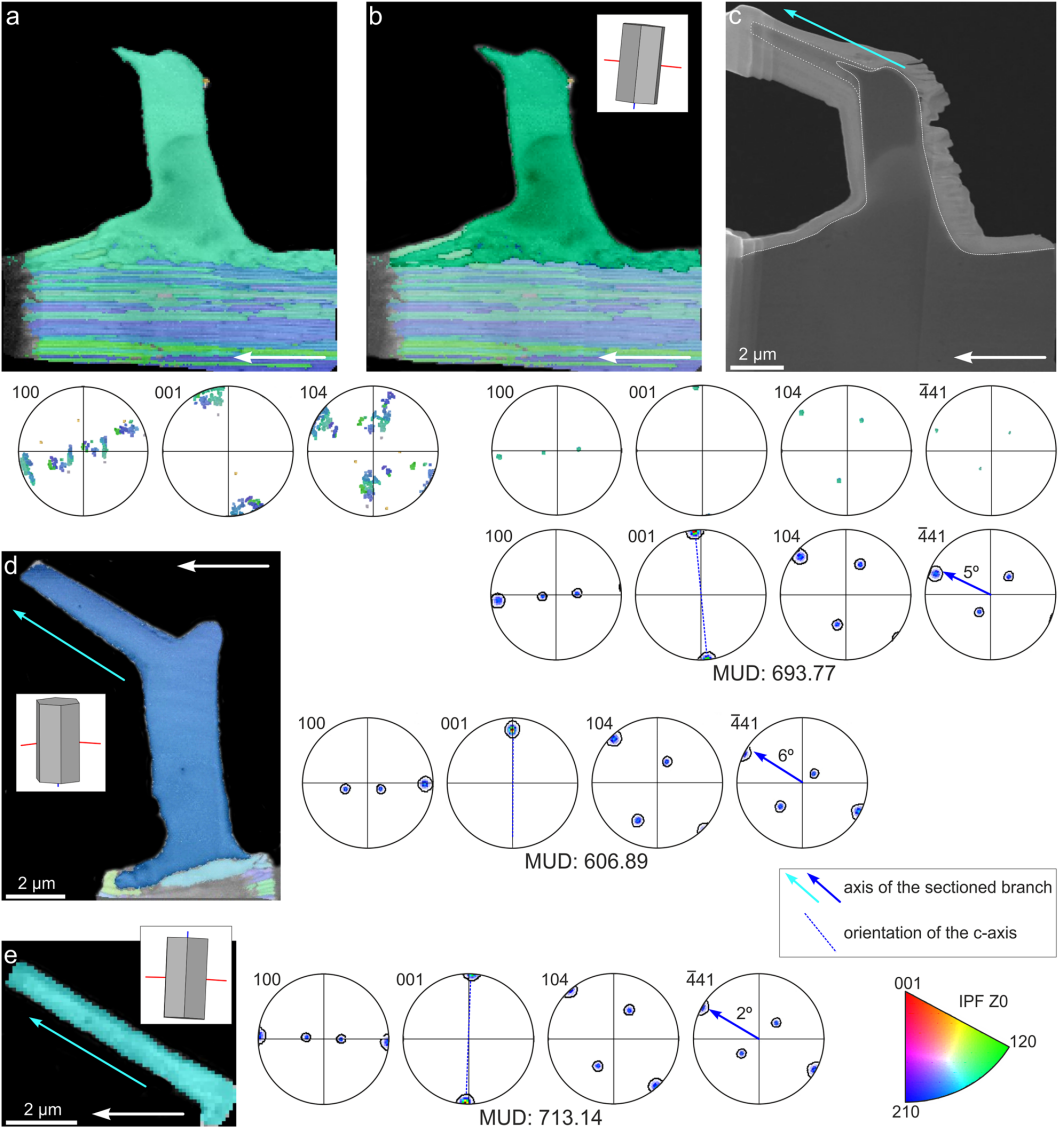
Crystallography of the second-order aerials and underlying prisms of *Catillopecten natalyae* analyzed by EBSD. (**a**) Orientation map and corresponding pole figures of an aerial and underlying foliated material. (**b**) Subset of the map in (**a**), where the orientation of the aerial has been selected. The corresponding cell lattice is shown. Note continuity with the topmost laminae of the foliated layer. The lower pole figures correspond to the subset. The top pole figures contain the raw data and the bottom ones are density plots. (**c**) STEM image of the FIB-prepared lamella, where the complete extension of the branch (axis indicated with arrow) can be seen. (**d**) Orientation map of a complete stem and branch. The main axis of the branch, the corresponding cell lattice, and representative pole figures are indicated. (**e**) Orientation map of an incomplete branch. The main axis of the branch, the corresponding cell lattice, and representative pole figures are indicated. The long white arrows indicate the shell growth direction. The deviation angle between the branch axis and the position of the − 441 poles is indicated in (**b**), (**d**) and (**e**). Colour key diagram (bottom right of figure) valid for all maps.

All EBSD phase maps, both on the lamellae prepared by FIB-SEM and on shell surfaces, indicate that the only mineral phase present is calcite (Supplementary Fig. S5). The three orientation maps made on FIB-SEM prepared lamellae reveal consistent crystallographic structure and coherent orientation of the aerials (Fig. 6). In all cases, the aerial consists of a single calcite crystal, with very little internal misorientation, as indicated by the consistent colors in the orientation maps, the clustering of maxima in the pole figures, and the high MUD values (> 600) (Fig. 6a, b, d, e). In the only case when the foliated material of the underlying prism was also mapped, there was crystallographic continuity between this and the aerial although the orientation of laths changes deeper within the prismatic-foliated layer (Fig. 6a). In the two most complete specimens, the stem is close in orientation to the c-axis of calcite (Fig. 6b, d). In all cases, the branch axis is at a high angle to the c-axis (estimated at 59, 58° and 62° in Fig. 6b, d, and e, respectively), and points in the direction to the {104} faces, although at a lower inclination to the 104 maxima (pole figures in Fig. 6b, d, e). In all cases, there is a good match between the elongation axes of the branches and the < − 441> directions, i.e. the edges of the calcite rhombohedron (deviation angles in each case are provided in Fig. 6). The constancy in orientation of both the stem and branches, arranged according to the three-fold symmetry of calcite, strongly argues for a crystallographic control on the morphology of the aerials.

The maps made directly on the surface (Supplementary Fig. S6), despite their relatively low quality, revealed a broad and consistent co-orientation of the prisms (MUD values around 80), with the 001 maximum slightly displaced in the growth direction of the shell, and one of the 104 maxima aligned with this same direction.

TEM indexations of the FIB-SEM prepared lamella shown in Fig. 6d fit in with the EBSD data (Supplementary Fig. S7a). The branch of the aerial pointing in the growth direction is also oriented in the direction of the (104) plane. A similar orientation is found in the foliated prismatic shell layer (Supplementary Fig. S7b). The superposed folia display small misorientations.

## Discussion

This study has shown that the remarkable aerial structures of two species of *Catillopecten* are mineralized intraperiostracal structures. They are the first such structures discovered to be made of calcite rather than aragonite and adopt far more sophisticated morphologies apparently under strict crystallographically determined control.

### Crystallography of the aerials and their relationship with the underlying shell

**Figure 7:**
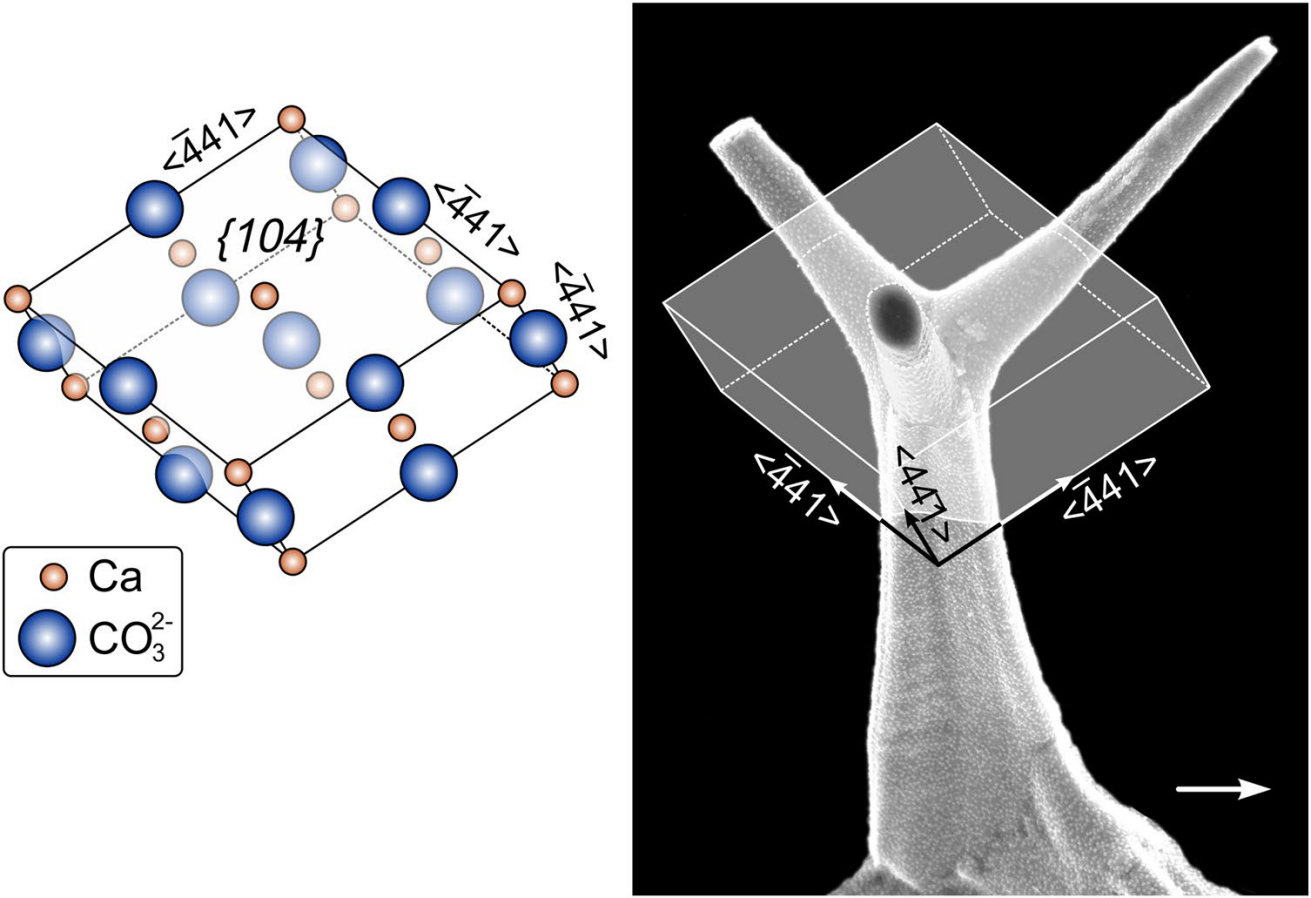
Crystallographic model for the second-order aerials of *Catillopecten*. The stem coincides with the c-axis (i.e. the line joining the upper and lower vertices of the {104} calcite rhombohedron, to the left), whereas the branches are aligned with the edges of the same rhombohedron (< − 441 > directions). The right sketch shows an aerial of *C. natalyae* partly enclosed within a rhombohedron, in approximate crystallographic coincidence. The arrow indicates the shell growth direction.

According to the crystallographic data the aerials are monocrystals that are in full crystallographic continuity with the top-most laminae of the prismatic layer. The high MUD values obtained (Fig. 6) are typical of inorganic crystals^20^, but rare in biogenic crystals (recorded only in aragonitic myostracal prisms^21^, and in the calcitic columnar prisms of terebratulid brachiopods^22^). The c-axes of the aerials are roughly parallel to the stem axis. According to the pole figures, the branches are at a high angle (58–62°) to the c-axis. The orientations of the branches match in with the <− 441> directions, i.e. the edges between the rhombohedron faces. In a theoretical calcite rhombohedron, these directions are at 63.64° to the c-axis, which is very close to the calculated values (between 58° and 62°). The approximately 4° difference between the samples analyzed (see estimated angle between the branch axis and the − 441 maximum in Fig. 6b, d, e) does not seem significant in view of the observed twisting and bending cases of the branches (Figs. 1, and 3g, h, l, m, and Supplementary Figs. S2 and S3). The <− 441> directions are favoured directions in calcite because they are the strongest periodic bond chains (PBCs)^23,24^, i.e. uninterrupted chains of strong bonds formed in the crystal lattice. Accordingly, they are the least prone to be inhibited by the presence of biomolecules in biocrystals. In the first-order aerials, the edges of the flanges would correspond to very narrow prismatic faces of the {010} type, able to grow uninhibited, contrary to the rest of prismatic faces. Each flange would contain one of the <− 441> PBCs. These morphologies resemble those of calcite crystals grown in the presence of highly charged proteins^25^. In the same line of argument, the frequently observed flat and smooth fracture surfaces of branches of second-order aerials (Fig. 3h, j, n) can be interpreted as {104} rhombohedral faces, which constitute the usual cleavage planes in calcite. This is supported by their persistent orientations and smoothness. All in all, it is clear that the morphology of the aerials is determined by crystallography. Our interpretation of this is shown in Fig. 7.

The EBSD data acquired on the shell surface, shows that the foliated prisms of the outer layer are co-oriented, with the c-axis (001 maximum) slightly inclined in the growth direction and one rhombohedral face (104 maximum) pointing in the growth direction (Supplementary Fig. S6). This orientation matches perfectly with previous data on the crystallography of the foliated microstructures of the orders Pectinida and Ostreida^26–28^, which is not surprising since the foliated material forming the outer shell layer of *Catillopecten*, and in the Propeamussiidae in general, even though made of particularly thin laths, must be classified within the same microstructure^29^. This material shows preferential growth along a direction that is at a low angle to the plane of the a-axes of calcite, and intermediate between two a-axes. Accordingly, the c-axis is at a high, though variable, angle to the growth direction. Since the laths also tend to dip at a low angle toward the shell growth margin, there is an overall co-orientation of the foliated material with respect to the shell margin. Thus, it is understandable that, even though the prisms of *Catillopecten* have independent origins, their constituting laths become broadly co-oriented with respect to the shell growth margin. Since aerials inherit their orientations from the underlying foliated prisms (i.e. they grow epitaxially onto the prisms), the overall orientation of the latter must dictate that of the aerials (Figs. 1, 2b, 3a–c, g–l, and 4d–f, and Supplementary Figs. S2 and S3). The strict co-orientation of aerials placed onto a single prism (Fig. 3k, l) also makes sense if every prism constitutes a crystallographic unit.

### Model for shell and aerial growth

**Figure 8:**
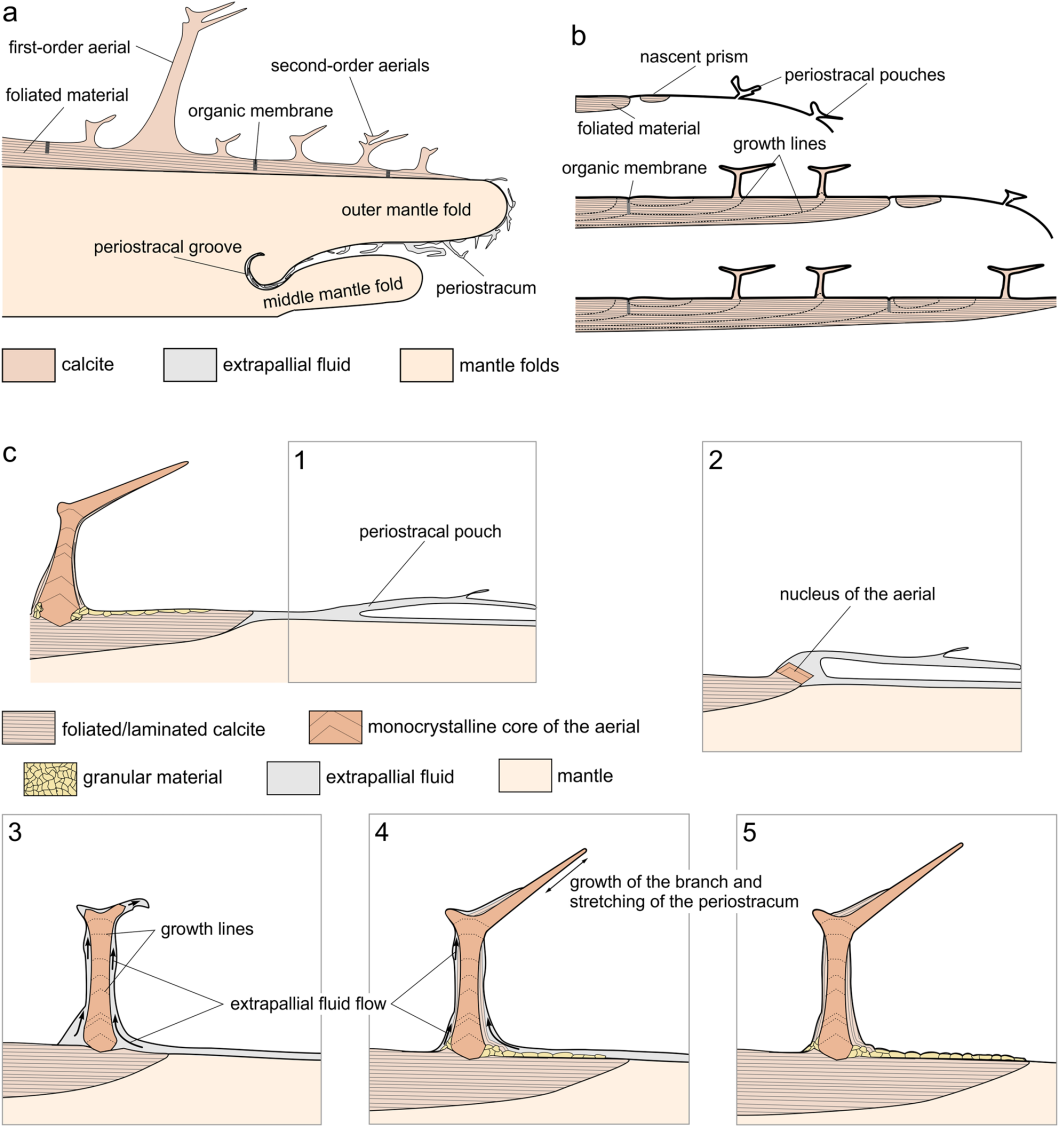
Model for the shell and aerial production by *Catillopecten*. (**a**) General scheme for the shell formation. The heavily folded periostracum is secreted within the periostracal groove and slides along the internal surface of the outer mantle fold. At the tip of the outer mantle fold, the periostracum is reflected toward the dorsum, and shell calcification begins, with the production of the outer prismatic layer. In the direction to the dorsum (to the left) aerials are produced in sequence on top of the shell, whereas a purely foliated layer is secreted below the prismatic layer. (**b**) Sequence of events (from top to bottom) for the production of prisms. Prisms initiate separated from each other. Upon meeting, they are initially separated by an organic membrane that later disappears in the direction toward the shell interior. (**c**) Stages (from 1 to 5) in the formation of the aerials. (1) Sliding of the periostracal pouch along the mantle surface. (2) Arrival of the periostracal pouch to the mineralization front, and formation of the initial nucleus of the aerial by the mantle. (3) Growth in height of the aerial when the mantle has abandoned the aerial production site. There is still supply of extrapallial fluid from the mantle in the passageway between the shell surface and the periostracum. (4) Aerial growth is almost complete, and production of laminated and granular deposits takes place at the voids between the periostracum and the monocrystalline core of the aerial. Conversely, the periostracum becomes stretched at the most elevated parts of the aerials, particularly, the branches. (5) Growth of the aerial (tips of the branches) is completed, as well as the production of laminated and granular deposits. The periostracum adheres to the surface of the aerial.

As in all bivalves, the first formed shell structure is the periostracum. This originates within the periostracal groove and is extruded towards the edge of the OMF (Figs. 5, and 8a). In *Catillopecten*, compared to other taxa previously studied^4^ there is a remarkably high degree of folding of the periostracum within the periostracal groove. At the tip of the OMF, the periostracum becomes reflected and shell secretion proceeds within the narrow space between the periostracum and the outer surface of the OMF (the extrapallial space). The centers of origin of prisms are dorsally placed but slightly displaced from the contact with the dorsal neighbor or neighbors (Fig. 2f, g), i.e. the prism initiates isolated from the previous prisms, although the contact is established very soon after (Fig. 8b). Upon contact, prims must already be placed onto the outer surface of the OMF since they already form a continuous, non-pliable shell plate (Fig. 8a).

Prism formation must pre-date that of the aerials (e.g. Figure 8b). These must initiate as soon as the mineralization front reaches their position (Fig. 8c, stages 1 and 2), as indicated by the lack of deflection of growth lines on the prism surface when they reach the dorsal side of the aerials (Figs. 2f, g, and 3m). Growth lines observed close to the base of the aerials (Figs. 3d, g, h, m, and Supplementary Fig. S3a) indicate that (1) the dorsal slope of the aerial stem is initiated first, (2) the mineralization margin must abandon the position of the aerial when this is in an incipient growth stage (Fig. 8c, stages 2 and 3). The distribution of growth lines visible on the surfaces of the stems (Figs. 3c–e, g, h, m, and Supplementary Fig. S3a), proves that later growth of the aerials proceeded steadily from the base of the stem toward the tips of the branches (Fig. 8c, stages 3 and 4). All in all, the mantle must “sow the seed” for every aerial, which then grows in isolation, in a sort of remote biomineralisation. Despite being remote products, the aerials display the nanoroughness typical of biominerals (Supplementary Fig. S4). This feature has been interpreted as resulting either from a particle attachment process^30,31^ or crystallization from an amorphous precursor^32,33^. Whatever the process, nanoroughness results from the intervention of biomolecules in the mineralization process. The development of threefold branches after the aerial acquires a certain height is particularly appealing. This could be due to a stronger inhibition of growth in all directions except the <− 441> directions due to organics.

Regarding aerial growth, there is the intriguing issue that the aerials are completely sheathed by periostracum, but do not pierce it upon growth. One hint comes from the fact that upon extrusion from the periostracal groove, the periostracum of *C. malyutinae* appears extremely folded and loose (Figs. 5, and 8a). The fold amplitude (Fig. 5) is comparable to the length of the stems of the aerials (Fig. 4c–g) in this species. We do not know the appearance of these folds in three dimensions but hypothesise that they constitute pre-fabricated pouches. In our model, aerials begin to grow when the periostracal pouch becomes positioned onto a prism at the mineralization front, as explained above (Fig. 8c, stage 2). In the context of the pre-fabricated pouch hypothesis, the alternation between first- and second-order aerials observed in *C. natalyae* must be pre-dated by the cyclical formation of first- and second-order periostracal pouches. How secretion of the periostracal pouches, and that of the aerial “seeds” by the mantle edge below them is co-ordinated, is unknown, but some sort of contact recognition of the periostracal pouches by the shell-forming mantle cells might exist. Contact recognition processes by the molluscan mantle cells have been invoked for the production of a series of microstructures^34–36^.

Later growth of the aerial within its periostracal pouch can be likened to a finger sliding into a wrinkled glove (Fig. 8c, stages 3 and 4). The looseness of the pouch must diminish as the aerial grows within it. The fit is never perfect, and the periostracum must remain loose in some areas and stretch in some others (Fig. 8c, stages 3 and 4). We interpret the laminated and granular deposits around the aerial solid core (Figs. 3d–h, and 4g–p) as infillings of void spaces left between the aerial core and the periostracal pouch (Fig. 8c, stage 4). Evidence of periostracal stretching at the branch tips comes from the low density of pimples usually observed (Figs. 3o, and Supplementary Fig. S3). The draped deposits observed at the bases of the first-order aerials are produced by lifting of the periostracum during growth in height of the aerials, and subsequent mineralization of the resulting void spaces (Figs. 1, 2b, and 3d-f, and Supplementary Fig. S2). Granular deposits on the shell surface (Figs. 2h, 3g, m, and 4n, p) and calcified periostracal wrinkles (Figs. 1, 2f, and 3f, and Supplementary Fig. S2) may have a similar origin (illustrated in Fig. 8c, stages 4 and 5).

It is striking that the final volume of the calcite forming the aerials (including core and crusts) is at least equal, if not higher, than that of the original periostracal pouch. This implies that there must be some supply of mineralizing super-saturated extrapallial fluid during growth of the aerial, even though this is out of the contact with the mantle. This may happen if the periostracum is not firmly attached to the shell, such that the mineralizing fluid can circulate in between and ascend along the aerial surfaces to nourish its distal parts (Fig. 8c, stages 3 and 4). This situation could not last for long since the entire growth of the aerial must be a fast process. The granular deposits observed on some extensions of the shell surface (Figs. 2h, 3g, m, and 4n, p) must be likewise formed within a space between the periostracum and the outer foliated prismatic layer proper (Fig. 8c, stages 3 to 5). Once calcification has finished and the extrapallial fluid has become depleted, the periostracum adhered tightly to the walls of the aerial (both the core and the secondary deposits) (Fig. 8c, stage 5).

### Final remarks

The aerials of the studied species of *Catillopecten* are monocrystals in which the stem and branches correspond to crystallographic directions, which have been selected for growth. Similarly shaped inorganic calcite crystals have been obtained by mixing calcium cations and carbonate anions in a silicate gel^37^, which were attributed to silicate ions being adsorbed on particular crystallographic planes, inhibiting their growth. In the case of *Catillopecten*, it is likely that a comparable role is played by organic molecules, able to inhibit and/or promote the preferential development of particular crystallographic faces and/or directions. The overall co-orientation of aerials is inherited from the underlying foliated layer.

The aerials of *Catillopecten* can be compared to the calcitic structures formed by other invertebrates, namely calcareous sponge spicules, octocoral sclerites and the larval sea urchin skeleton. Despite the beguiling similarities of these, we note that all are formed in close association with cells whereas the aerials described herein are remote. Calcite spicules of calcareous sponges are produced by specialized cells, the sclerocytes, in an extracellular space^38^. Their axes also follow crystallographic directions^39,40^, but in no case, the growth along <− 441> takes place. Octocoral sclerites have varied morphologies and are produced by calcifying cells called scleroblasts^41^, following a two-step formation process: intracellular and extracellular. They usually have a central axis, and a series of radial arms, which, in some instances, follow crystallographic directions. For example, the dumbbell-shaped sclerites of the gorgonacean octocoral *Corallium rubrum* have their long axes and arms along the <001> and <− 441> directions, respectively^42^, in a way similar to the stem and arms of *Catillopecten* aerials. The larval sea urchin skeleton is formed by primary mesenchyme cells in the embryo blastocoel^43^. It initiates from an initial micrometric calcite rhombohedron. Its branches also resemble the aerial branches, though they extend along the a-axes of calcite^44^.

All in all, the aerials of *Catillopecten* constitute a unique case of extremely sophisticated microornament which, once initiated by the cells of the mantle, have a morphology and co-orientation which is strictly determined by crystal growth laws.

## Material and methods

### Material

The material of *C. natalyae* and *C. maluytinae* was collected by the German-Russian deep-sea expeditions KuramBio (Kuril Kamchatka Biodiversity Study) (July 21st–September 7th, 2012) and KuramBio II (August 16–September 26, 2016) from the abyssal plain adjacent to the Kuril-Kamchatka Trench (Pacific Ocean) using an epibenthic sledge^15,45–48^. All specimens were preserved in 96% ethanol. We have used images and observations made in the description of *Catillopecten* species^15^ and used a further three individuals of each species (Supplementary Table S1) to conduct a more detailed investigation of the shell microstructure using the battery of techniques described below.

### Optical microscopy

One specimen of *C. malyutinae* was dehydrated and embedded in Technovit 7200 VLC methacrylate-based resin in five steps. The first three steps were combinations of ethanol (Et) and progressively increasing proportions of Technovit (T) (30 T: 70Et; 50 T: 50Et; 70 T: 30Et); the last two steps consisted only of Technovit 7200 VLC. Samples were then polymerised. Embedded specimens were dorsoventrally sectioned to thicknesses of 50 and 10 μm using the EXAKT 300CL cutting band system. All sections were stained with toluidine blue (1%). They were observed with an Olympus VS120 microscope. The entire process was carried out in the Andalusian Centre of Nanomedicine and Biotechnology (BIONAND, Málaga, Spain).

### Scanning electron microscopy (SEM) and atomic force microscopy (AFM)

To study the surface morphology of the aerials, initial observations were made on carbon-coated material using the Field Emission SEMs (FESEM) Zeiss SIGMA 300 VP (A.V. Zhirmunsky National Scientific Center of Marine Biology, Far Eastern Branch, Russian Academy of Sciences, Vladivostok, Russia) as part of Kamenev (2018), FEI QemScan 650F (University of Cambridge, UK), Zeiss Auriga, FEI QemScan 650F and TESCAN Amber X of the Centre for Scientific Instrumentation (CIC) of the University of Granada (UGR), Spain. These investigations were sometimes hampered by debris adhering to the surface of the fragile valves. Although some dried shell fragments of *C. natalyae* were ultrasonicated we realised that this procedure removed most aerials. Instead, specimens were cleaned by light brushing only.

The image in Supplementary Fig. S2 is a stereoscopic image (anaglyph) created by overlapping two SEM micrographs recorded from different perspectives. A SEM micrograph was acquired at a tilt angle of 0°, while the other was acquired after tilting the specimen by 8° along a vertical axis (i.e. perpendicular to the axis joining both eyes of the observer). An eucentric point was used, which enabled tilting without introducing any lateral translation. By merging both images, a three-dimensional effect of the imaged area is obtained. Both SEM micrographs were obtained in the FEI QemScan 650F FESEM (CIC, UGR). The two images were merged and read into the xT microscope Control software (FEI, Hillsboro, OR, USA), turned into red and cyan images and overlapped using the "stereo pair" option. The 3D effect is fully appreciated by observing them with easily obtainable red/cyan glasses.

Study of the internal microstructures by SEM and AFM of the aerials was made for the methacrylate-embedded specimen of *C. malyutinae* (see optical microscopy, above). The sample was polished with a polycrystalline diamond suspension (Struers, 1 µm and 0.25 µm), using high-density wool felt pads (Hi-Tech Diamond polishing machine, All-U-Need model). SEM observations were done with the FEI QemScan 650F (CIC, UGR). For AFM we used the Park Systems NX20 (CIC, UGR) equipment with a cantilever MikroMasch ACTA (K = 40 N/m, F = 320 kHz). We used tapping mode to record height, amplitude, and phase signals. Images were obtained with Smart Scan v12 and processed with XEI software (4.3.0. Build2, Park System).

### Transmission electron microscopy (TEM)

One specimen of *C. maluytinae* with attached soft-parts was fully decalcified by immersion in 4% EDTA for several days. Pieces of the mantle, with their associated periostraca, were excised mainly from the ventral areas. They were CO_2_-critical-point dried (Polaron CPD 7501), post-fixed in OsO_4_ (2%) for 2 h at 4 °C and embedded in epoxy resin (Aname Epon 812). Blocks prepared in this way were sectioned with an ultramicrotome LEICA Ultracut R and prepared following standard procedures. Ultrathin sections (50 nm) were stained with uranyl acetate (1%) followed by lead citrate. They were observed with TEM (Zeiss Libra 120 Plus) at the Center for Scientific Instrumentation (CIC) of the University of Granada. Due to shortage of material, no specimen of *C. natalyae* was available for TEM observation.

The two most complete lamellae prepared by FIB and used for EBSD (see below) were also observed using a double Cs corrected FEI Titan G2 60–300 TEM equipped with an X-field emission gun and a Gatan Ultrascan camera (CIC, UGR). Imaging was performed in TEM mode at 300 kV, and 0.5–1 s exposure.

### Electron backscatter diffraction (EBSD)

Three lamellae (~ 100 nm thick) of smaller aerials of *C. natalyae* were prepared by means of FIB-SEM. The aerials were sectioned along the branch pointing in the local growth direction of the shell and the stem. One of the lamellae contained also laths of the underlying foliated layer. Another lamella broke during the process, losing the stem, and only the branch remained, although it also provided relevant information. The lamellae were mapped by scanning transmission electron microscopy coupled to transmission Kikuchi diffraction (STEM-TKD), at an acceleration voltage of 30 kV, and at step sizes of 99 and 30 nm, depending on the desired resolution. Three additional maps were made directly on the intact surface of the shell. Due to surface irregularities, the quality of these maps was deficient, but nevertheless allowed us to estimate the degree of co-orientation of the prisms. Step size was 0.1 µm in all cases, and voltage was 30 kV. All the procedure was carried out in a Zeiss Auriga CrossBeam Workstation, equipped with an Oxford Instruments Nordlysnano EBSD detector and a Cobra FIB Column, belonging to the Center for Research, Technology and Innovation of the University of Sevilla (CITIUS).

Information obtained from EBSD measurements is provided as phase maps, grey-scaled band contrast images, and color-coded crystal orientation maps. The texture is shown in the form of individual data points or contour pole figures. In the latter case, we use the lowest possible degree for half-width (5°) and cluster size (3°). As usual for calcite, we provide 100, 001 and 104 pole figures. Crystal co-orientation measurements are derived from density distributions in pole figures and are given as MUD (multiple of uniform distribution) values. The higher the MUD, the higher the co-orientation. All post-processing was done with the Oxford Instruments CHANNEL 5 HKL software.

## Supplementary Information

Supplementary Information.

## Data Availability

All data generated and/or analyzed during the current study are available upon request by contact the corresponding author. The crystallographic (EBSD) datasets are available in the Zenodo repository, https://zenodo.org/record/6375610#.YjmqtzWCGUk.
