# Supplementary material for: Crystallographic control of the fabrication of an extremely sophisticated shell surface microornament in the glass scallop Catillopecten
Source: Sci Rep. 2022 Jul 7;12:11510. doi: 10.1038/s41598-022-15796-1 (PMC9262964; doi:10.1038/s41598-022-15796-1)
Supplement: Supplementary file 1 — Supplementary Information. [file 41598_2022_15796_MOESM1_ESM.pdf]

Crystallographic control of the fabrication of an extremely sophisticated shell surface microornament in the glass scallop *Catillopecten*

Antonio G. Checa[1,2], Carmen Salas[3], Francisco M. Varela-Feria[4], Alejandro B. Rodríguez-Navarro[5], Christian Grenier[1], Gennady M. Kamenev[6], Elizabeth M. Harper[7]

[1]Department of Stratigraphy and Paleontology, University of Granada, 18071 Granada, Spain
[2]Instituto Andaluz de Ciencias de la Tierra, CSIC-Universidad de Granada, 18100 Armilla, Spain
[3]Department of Animal Biology, University of Málaga, 29071 Málaga, Spain
[4]Centro de Investigación, Tecnología e Innovación, Universidad de Sevilla, 41012 Sevilla, Spain
[5]Department of Mineralogy and Petrology, University of Granada, 18071 Granada, Spain
[6]A.V. Zhirmunsky National Scientific Center of Marine Biology, Far Eastern Branch, Russian Academy of Sciences, Vladivostok 690041, Russia
[7]Department of Earth Sciences, University of Cambridge, Downing Street, Cambridge, CB2 3EQ, UK

Supplementary Figures S1 to S7 and Supplementary Table S1

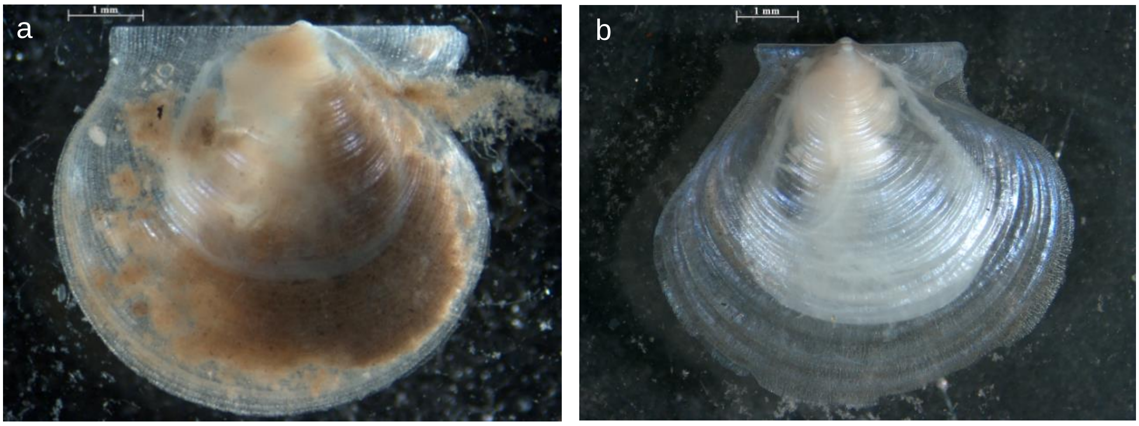

Figure S1. Holotypes of both species of *Catillopecten*. (**a**) *Catillopecten natalyae* Kamenev, 2017 (MIMB 34150) (exterior view of right valve, shell length 6.0 mm); abyssal plain adjacent to the Kuril-Kamchatka Trench, Pacific Ocean (43°02.31′ N, 152°59.16′ E), 5,222 m, box corer, Coll. G.M. Kamenev, 16.08.2012 (RV Sonne, cruise no. 223, stn. 7-4). (**b**) *Catillopecten malyutinae* Kamenev, 2017 (MIMB 34148) (exterior view of right valve, shell length 7.1 mm); abyssal plain adjacent to Kuril-Kamchatka Trench, Pacific Ocean, (42°14,69′N, 151°44,05′E – 42°14,26′N, 151°42,49′E), 5,127 m, epibenthic sledge, Coll. A. Brandt, 20-VIII-2012 (RV Sonne, cruise no. 223, stn. 8-9).

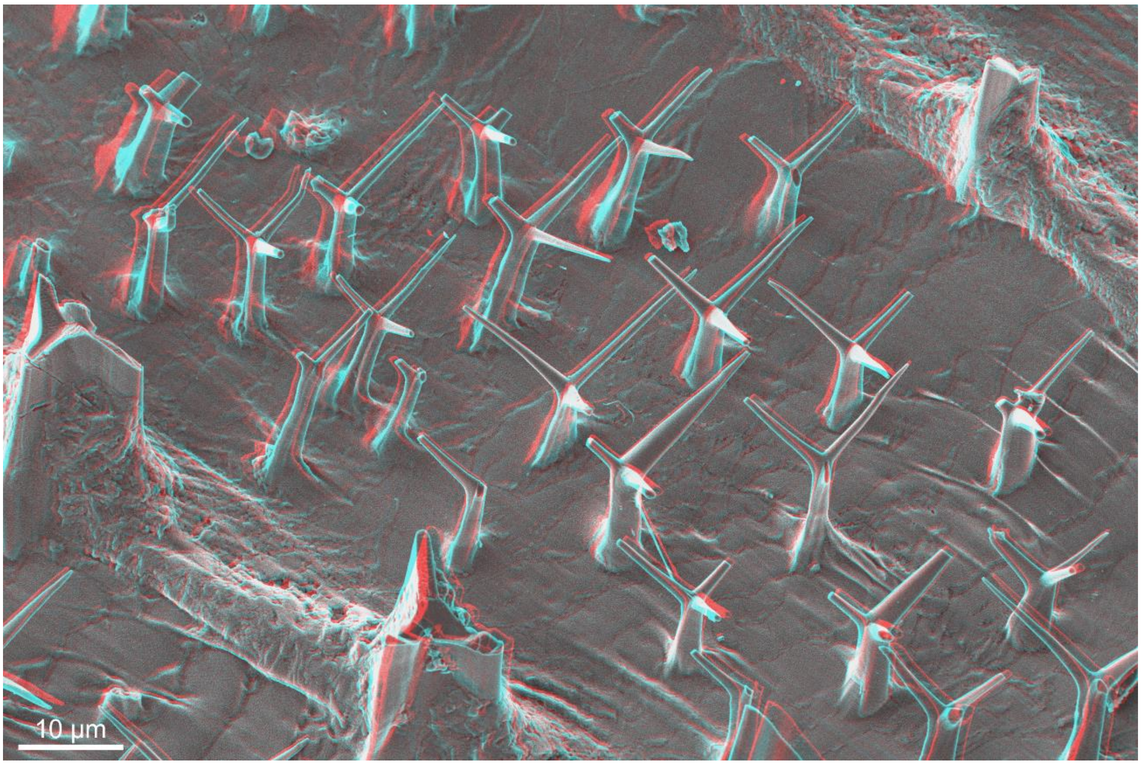

Figure S2. Anaglyph of an area of the shell surface of *Catillopecten natalyae* (same as in Fig. 1), comprising two ribs with broken first-order aerials and an inter-rib area with second-order aerials. To appreciate the 3D effect, red-cyan glasses have to be used.

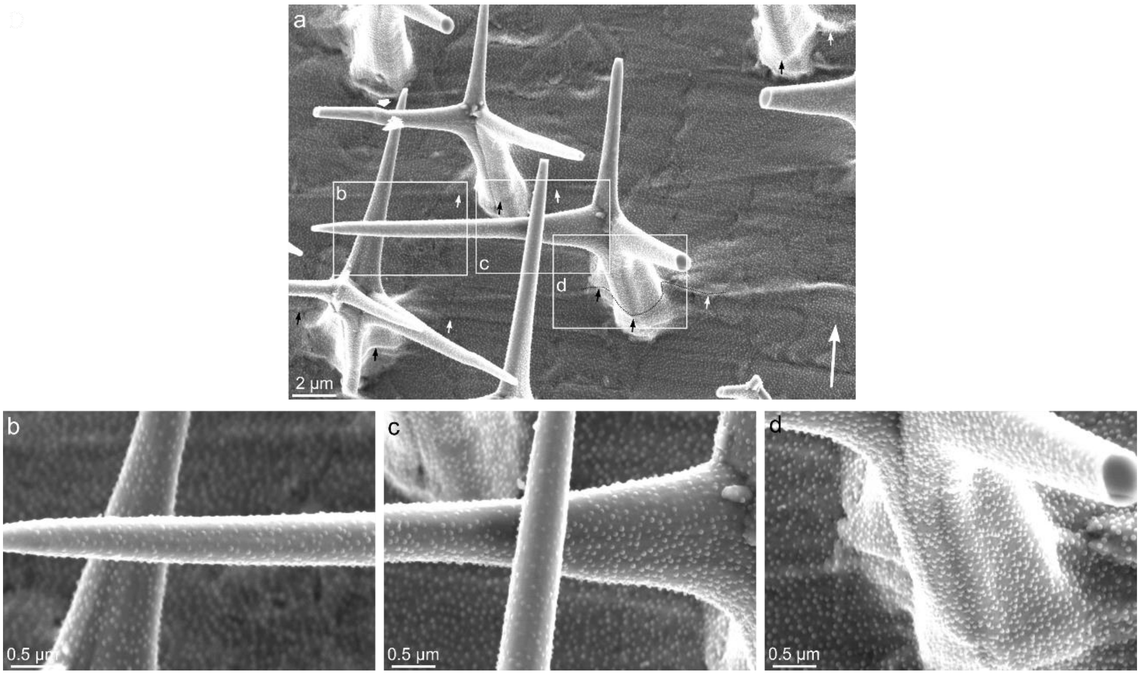

Figure S3. Second-order aerials of *Catillopecten natalyae*. (**a**) Group of several individuals. Small thin arrows point to growth lines close to the base of the aerials; one growth line has been outlined with broken line; the wide small arrow indicates a locally twisted branch; the long arrow points in the growth direction of the shell. (**b**-**d**) Details of areas of the central aerial in A (frames) showing decreasing density of periostracal pimples from the base of the stem (**d**) to the tips of the branches (**c**, **b**).

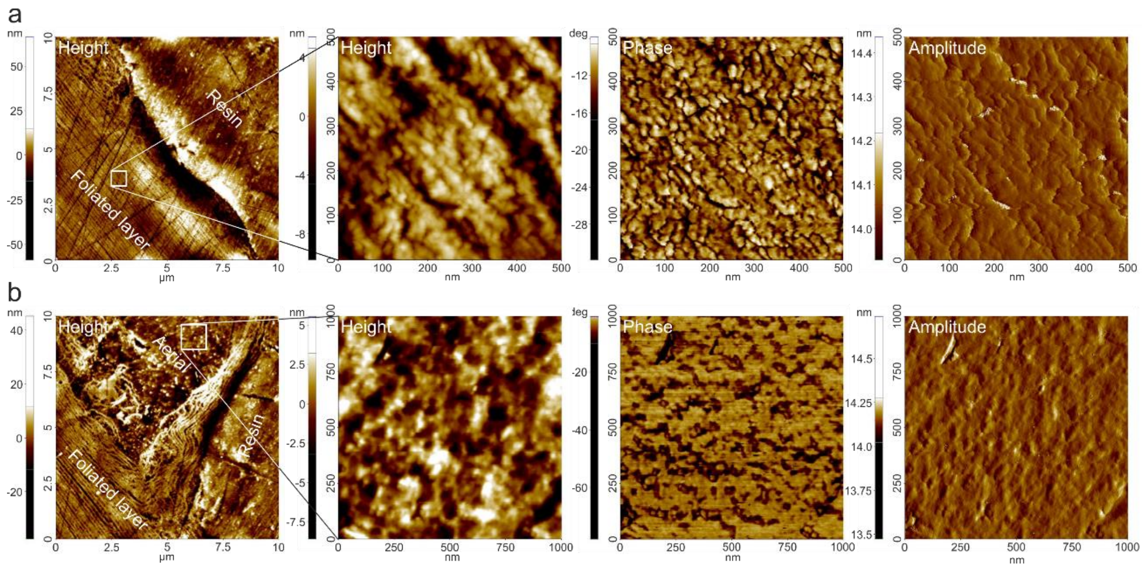

Figure S4. AFM analysis of the foliated layer (**a**) and the interior of the aerials (**b**) of *C. malyutinae*. The left images are low magnification views with indication of the areas examined. The three signals (height, phase, amplitude) reveal a clear nanoroughness of both materials. Same sample as in Fig. 4g.

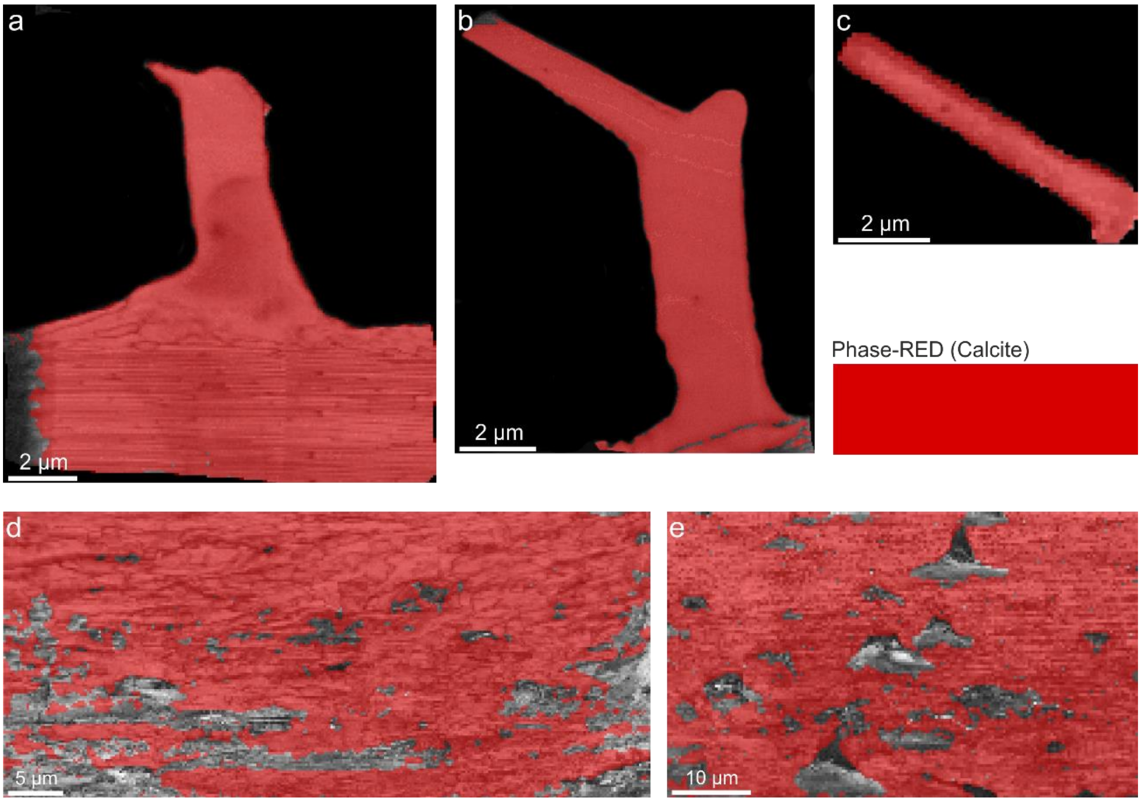

Figure S5. Phase maps corresponding to the orientations maps in Figs. 6a, d, e, and Supplementary Fig. S6a, b (**a** to **e**, respectively).

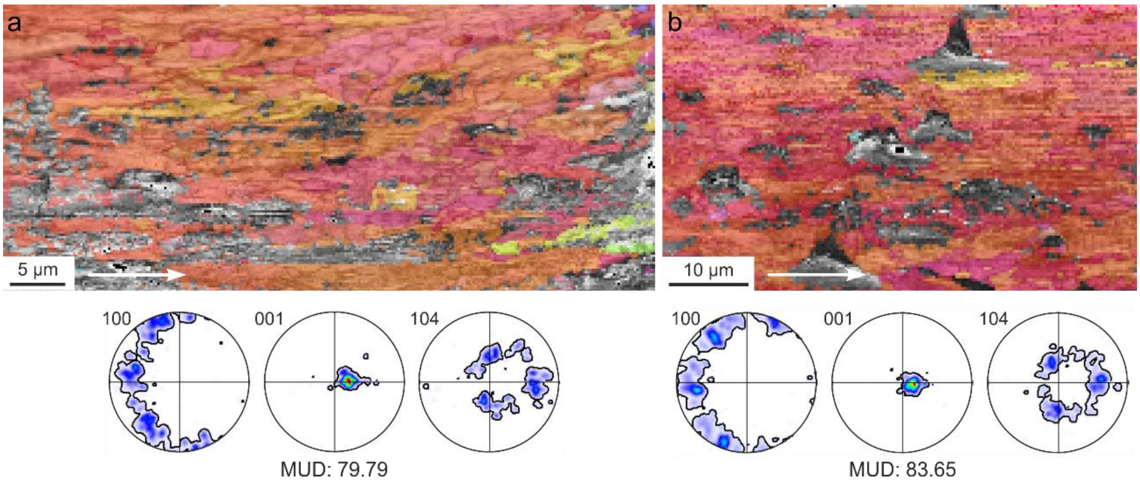

Figure S6. EBSD maps of the outer shell surface of *Catillopecten natalyae*. (**a**, **b**) Orientation color maps superposed to the corresponding image quality maps. Despite the low quality of the maps, the pole figures indicate a good coorientation of the foliated prisms, with the c-axis (100 maximum) slightly inclined in the growth direction (arrows), and one 104 maximum looking in that direction.

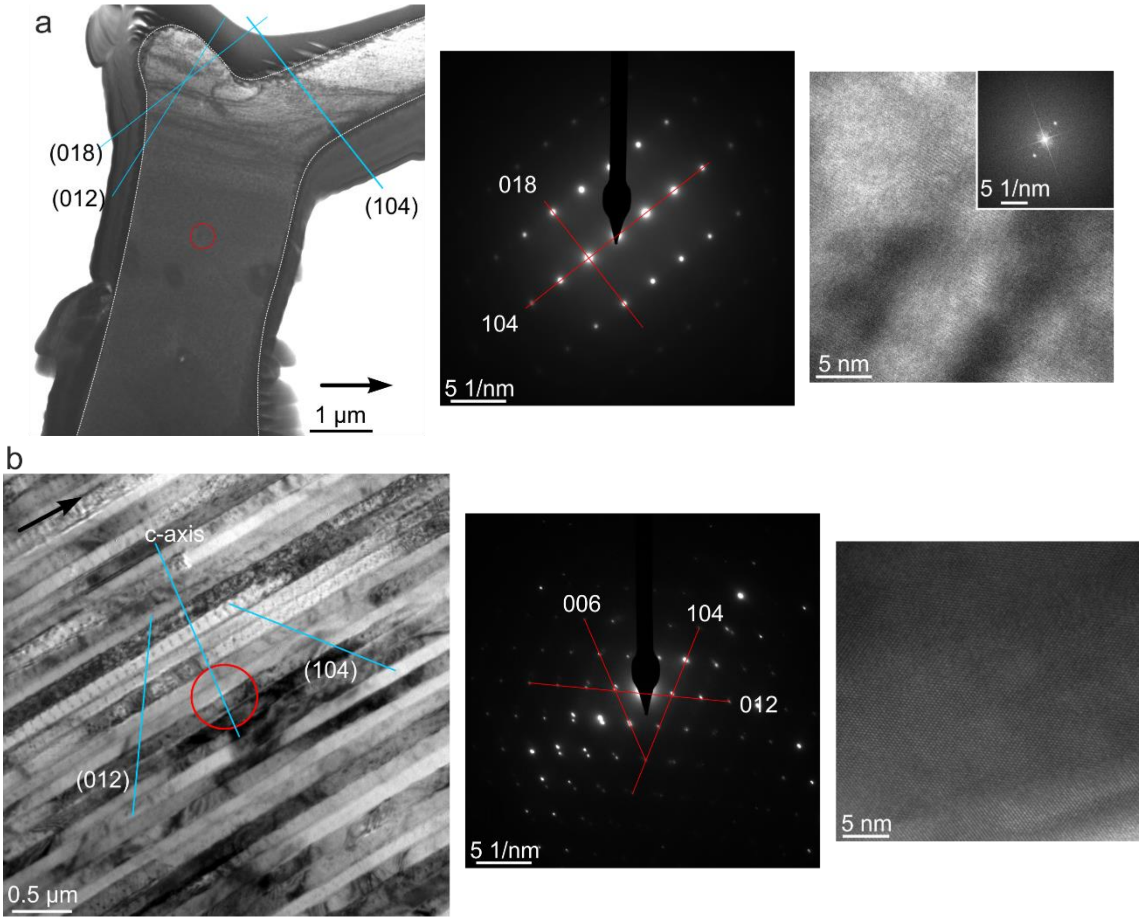

Figure S7. TEM imaging and analysis of a FIB-prepared lamella of *Catillopecten natalyae*. (**a**) TEM image of the sample (same as in Fig. 6d) at low magnification showing the traces of relevant calcite crystal faces (104), (012) and (018), deduced from SAED patterns collected at slightly different tilting, viewed along zone axes [-4-81] (center) or [-4-21] (right). The SAED patterns display calcite single crystal patterns indicating that the arms have a coherent crystallographic orientation and the material behaves as a single calcite crystal as shown also in the high-resolution image and associated FFT pattern (showing only 104 spots) (right image). (**b**) TEM image of the underlying foliated prismatic layer (left), showing very thin lamellae (about 130 nm thick) with very uniform thickness and orientation (the c-axis is almost perpendicular to the foliation). The SAED pattern (center) shows double spots from superimposed calcite single crystal patterns due to a small misorientation between adjacent lamellae (3.4° between the two encircled lamellae in the left image). The high-resolution image (right) confirms the single crystalline nature of lamellae with a very coherent orientation. The SAED pattern (central image) and high-resolution images (right images) confirm that each lamella has a coherent orientation and behaves like a single crystal.

Table S1. Material of *Catillopecten* species examined in the present study. N, number of live specimens.

Species	Ship/cruise	Date	Start		End		Depth (m)	N
			Latitude N	Longitude E	Latitude N	Longitude E		
C. natalyae	*Sonne* 223	23.08.2012	40°35.49'	150°59.92'	40°34.25'	150°59.91'	5399-5398	2
	Sonne 250	19.08.2016	43°48.593'	151°46.433'	43°48.598'	151°46.477'	5107	1
C. malyutinae	*Sonne* 250	20.08.2016	43°48.602'	151°47.124'	43°48.455'	151°47.171'	5352-5104	3
